# From Cognitive MR-Targeted Fusion Prostate Biopsy to Radical Prostatectomy: Incidence and Predictors of Gleason Grade Group Upgrading in a Chinese Cohort

**DOI:** 10.1155/2022/7944342

**Published:** 2022-08-17

**Authors:** Huaqing Yan, Yiming Wu, Xiaobo Cui, Sinian Zheng, Peng Zhang, Rubing Li

**Affiliations:** Department of Urology, Ningbo Medical Center Lihuili Hospital, Ningbo, Zhejiang 315000, China

## Abstract

**Purpose:**

To access the incidence and predictors of Gleason grade group upgrading from cognitive MR-targeted fusion prostate biopsy to radical prostatectomy in a Chinese cohort.

**Materials and Methods:**

We included 199 patients in our institution between January 2016 and June 2021. Multivariable logistic regression model and nomograms were utilized to analyze the collected data.

**Results:**

The concordance rate of biopsy Gleason grade group and radical prostatectomy was 50.3% (100 in 199). Upgrading occurred in 80 (40.2%) patients and 37 (68.5%) patients have an upgrading Gleason grade group when the biopsy Gleason grade group was 1. Multivariable logistic regression models were established to analyze the incidence and predictors of Gleason grade group upgrading from cognitive MR-targeted fusion prostate biopsy to radical prostatectomy. Biopsy Gleason grade group, prostate volume, and patient year were confirmed to be individual predictors of upgrading. Based on the logistic regression models, nomograms for predicting probability of prostate Gleason grade group upgrading were generated.

**Conclusions:**

We established a logistic regression model to predict the accuracy of prostate biopsy GG and provide the probability of upgrading. Clinicians should be more cautious when deciding the treatment strategy especially for prostate cancer biopsy GG1 patients. Future studies should expand the sample size and include more variables to improve the accuracy of predicting upgrading and prostate cancer early screening program is urgently needed in our city in China.

## 1. Introduction

Prostate cancer (PCa) is a common malignant cancer in elderly men with the highest incidence rate and second death rate in the United States [1]. According to estimates, there are 248530 new PCa cases and 34130 deaths in 2021 [1]. On account of the high prevalence of PCa worldwide, it is important to diagnose PCa early and evaluate the conditions of prognosis. The diagnosis of PCa before surgery relies on prostate biopsy. After diagnosing, PCa patients may undergo watchful waiting, active surveilling, external beam radiotherapy, brachytherapy, and radical prostatectomy.

The serum PSA, Gleason grade group, and clinical stage play the most important part in the process of treatment strategy generation for prostate cancer patients before surgery. However, it is reported that PSA level could be affected by a series of clinical factors such as tobacco use [2]. The Gleason grade group (GG) of prostate biopsy plays a critical part in the decision-making of treatment. Especially for patients who may not undergo surgery, the GG of prostate biopsy remains the most significant part for treatment decision and prognosis. Besides, the GG of prostate biopsy plays a key role in surgical operation such as intra-fascial prostatectomy and pelvic lymph node dissection. However, GG inconsistency accounting for upgrades still remains an important clinical issue. It is reported that only 40%-60% of prostate biopsy GGs were consistent with the final prostatectomy [3–10]. Due to the high discrepancy rate from biopsy to prostatectomy, it is urgent for physicians to predict prostate biopsy GG upgrades before surgery.

In recent years, MR-targeted prostate biopsy was reported to be superior to standard transrectal ultrasonography-guided biopsy for detecting clinically significant PCa [11]. Meanwhile, MRI test was recommended for patients before prostate biopsy. Emerging evidence has shown the importance of radiomics and its potential for personalized treatment and future applications [12]. We noticed that previous studies rarely focus on the GG inconsistency of patients who underwent MRI before biopsy. So, we aimed to evaluate the incidence and predictors associated with Gleason grade group upgrading from cognitive MR-targeted fusion prostate biopsy to radical prostatectomy using logistic regression model in a Chinese cohort. This may help physicians improve the accuracy of PCa diagnosis and provide a more precise treatment for patients.

## 2. Materials and Methods

Patient data was acquired from a prospectively collected database of PCa patients in The Ningbo Medical Center Lihuili Hospital. All included patients underwent a multiparametric magnetic resonance imaging (mpMRI) test before prostate biopsy. The cognitive MR-targeted fusion prostate biopsy was conducted with the guidance of transrectal ultrasonography and was performed transrectally by an ultrasonologist and a urologist with over 5 years of clinical experience. First, a traditional systematic 12-core transrectal biopsy was conducted. Then, a 2-core targeted biopsy was conducted aiming the suspicious area according to the understanding and experience of reading mpMRI and TRUS imaging in real time.

All of the included patients underwent laparoscopic radical prostatectomy after biopsy in our institution. The operation was conducted by a surgeon with over 10 years of experience. The prostate Gleason score (GS) was confirmed by the pathology experts in The Ningbo Pathology Center according to the Gleason score system. The pathologist who analyzed the samples is the same between the biopsy and the prostatectomy. We further transferred the Gleason score to grade group (GG): GS ≤6 (GG1), GS3+4 (GG2), GS4+3 (GG3), GS8 (GG4), and GS ≥9 (GG5) [13, 14]. Immunohistochemistry was done in the histopathological examination.

The following information was collected and involved in the analysis: the age of patient when diagnosing PCa; the GG (or Gleason score) of prostate biopsy and radical prostatectomy; the last tPSA before biopsy (≤1 month); number of positive cores and total cores; prostate volume; clinical stage; the existence of MRI-visible prostate lesions. All patients underwent a prostate MRI test before biopsy. The exclusion criteria of our study were as follows: (a) not enough clinical data; (b) hormonal therapy or neoadjuvant chemotherapy before surgery; (c) a history of TURP; (d) the patients with secondary biopsy. Thus, we included a total of 199 patients between January 2016 and June 2021.

The upgrading was defined: the GG of radical prostatectomy was higher than prostate biopsy; conversely was the downgrading. The clinical stage was evaluated according to AJCC Eighth Edition of the Tumor-Node-Metastasis Staging Classification before surgery according to the MRI result, whole body bone scan result, and prostate biopsy result [15].

Multivariable logistic regression model and nomograms were utilized to analyze the collected data. First, all variables were included in the initial model. Then, the variables that were not significant (*p* > 0.05) were removed individually to reach the final model. The Hosmer-Lemeshow test was conducted to examine the goodness of fit and *p* > 0.05 was regraded to be acceptable. The ROC curve and AUC were generated for the final model. Finally, nomograms were developed from the logistic regression models. The traditional statistical analysis was performed by IBM SPSS Statistic 24 and the nomograms were achieved by R version 3.5.3 (R foundation for Statistical Computing, Vienna, Austria).

## 3. Results

The baseline data of included patients are given in Table 1. The concordance rate of biopsy GG and radical prostatectomy GG was 50.3% (100 in 199). Upgrading occurred in 80 patients (40.2%) and detailed data of GG is provided in Table 2. Particularly, 68.5% (37 in 54) patients have an upgrading GG when the biopsy GG was 1.

**Figure 1:**
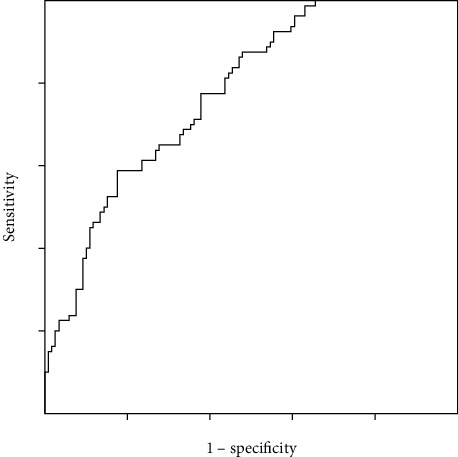
The ROC curve of the logistic regression model. The AUC of the model was 0.775 (95% CI 0.712-0.839).

**Figure 2:**
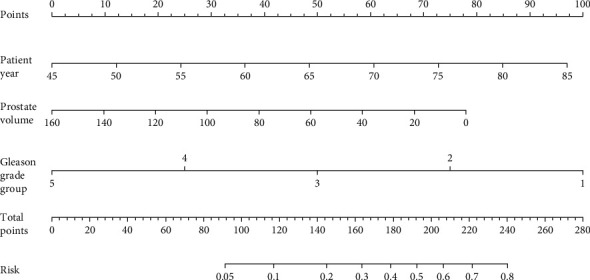
Nomograms for predicting prostate Gleason grade group upgrading. Instructions: To reach the predicted probability, locate the patient data at each axis and draw a vertical line to the “Points” axis and read the values. Sum all the points. Locate the sum to the “Total Points” axis and to draw a vertical line to the “Risk” axis to obtain the probability of upgrading or downgrading.

Multivariable logistic regression models were established to analyze the incidence and predictors of GG upgrading from prostate biopsy to radical prostatectomy. Biopsy GG, PV, and patient year were confirmed to be individual predictors of GG upgrading (details in Table 3). The result of the Hosmer-Lemeshow test was *p* = 0.403. The ROC curve was drawn and the AUC was 0.775 with 95% CI 0.712-0.839 (Figure 1). Based on the logistic regression models, nomograms for predicting probability of prostate GG upgrading were generated (Figure 2).

## 4. Discussion

Our study was aimed to evaluate the prostate grade group concordance of cognitive MR-targeted fusion biopsy with radical prostatectomy and predict the probability of upgrading in a Chinese cohort. For patients diagnosed with PCa via prostate biopsy, the GG of biopsy is critical for physicians to evaluate the condition of cancer and make decisions for the subsequent treatment, especially for patients who may not undergo surgery. Our study also helps clinicians to reduce the probability of underestimation of PCa and establish a more accurate surgery scheme such as intra-fascial prostatectomy and pelvic lymph node dissection.

We successfully constructed a multivariable logistic regression model for accurately predicting the probability of upgrading from prostate biopsy to radical prostatectomy and the final model was acceptable (AUC>0.7, the Hosmer-Lemeshow test *p* > 0.05). This result indicated that our model has the potential for improving the accuracy of PCa diagnosis and provides a more precise treatment for patients. We also did nomograms to help clinicians evaluate the risk of upgrading in a specific patient (Figure 2).

The result of prostate biopsy may not be accurate mainly because of sampling error, heterogeneity of tumor, and pathology error. In our study, the GG inconsistency rate from biopsy to radical prostatectomy was 49.7%. In particular, 68.5% of GG1 patients upgrades to higher GGs. Previous studies indicated that 30-50% of GG1 patients may upgrade [3, 8, 16–18]. The rate of upgrading in GG1 patients in our cohort was significantly higher than previously reported, which means that clinicians should be more cautious when deciding the treatment strategy for clinically low-risk PCa patients. Because GG1 was an essential condition for low-risk PCa patients, we screened the low-risk PCa patients before surgery. Only 15 patients in our cohort meet the conditions of low-risk PCa before surgery (PSA <10 ng/ml, prostate biopsy GG1, and clinical stage <T2b). Finally, 6 patients (40%) upgraded to higher Gleason grade group after surgery in 15 low-risk PCa patients. Actually, only 9 patients meet the criterion of low-risk PCa in our cohort (199 PCa patients, 4.5%). The limited amount of low-risk PCa patients impressed us that the PCa early screening program is urgently needed in our city in China.

Though the inconsistency rate was high, the risk factors of upgrading remain controversial: age, PSA, PSAD, prostate volume, number of positive cores, and a series of variables were predicted to be independent risk factors in different studies. Our study used the logistic regression model to include and analyze all available variables to achieve a more accurate model predicting upgrading. Provided the 9 variables mentioned in our method part, our model can calculate the probability of upgrading with high reliability and will be available for providing personalized prognostic information. Biopsy GG, PV, and patient year were confirmed to be individual predictors of GG upgrading in our study. We believe PV is important for prostate cancer patient because it affects the PSA density, another prognostic marker. Previous study reported that older age was associated with a significant increased risk of upgrading in a meta-analysis including 84296 patients [19]. Thus, both PV and patient year are convincing clinical indicators as the individual predictors of GG upgrading and may further affect the prognosis of prostate cancer patient,

There are some deficiencies in our study: (a) Our study was a retrospective, single-institution study which means that selection bias was unavoidable and this study type has some inherent disadvantages; (b) some variables, for example, total core percentage and the digital rectal examination result of patient, were not included in our model because these clinical data was missing. Meanwhile, some patients may take 5*α* reductase inhibitor and some patients may have urinary catheterization. We could not eliminate the influence of these conditions because of the lack of clinical data. Although PIRADS score may be related with GG upgrading, we felt regretful that some image of MRI was missing and the PIRADS score was inaccessible in our study [20].

## 5. Conclusion

We established a logistic regression model to predict the accuracy of prostate biopsy GG and provide the probability of upgrading. Clinicians should be more cautious when deciding the treatment strategy especially for PCa biopsy GG1 patients. Future studies should expand the sample size and include more variables to improve the accuracy of predicting upgrading and PCa early screening program is urgently needed in our city in China.

## Figures and Tables

**Table 1:** Baseline characteristics of included patients.

Age, mean ± SD (years)	68.82 ± 6.45
PSA, mean ± SD (ng/ml)	19.00 ± 23.72
Prostate volume, mean ± SD (ml)	38.55 ± 22.06
PSAD, mean ± SD	0.67 ± 1.28
Percent of positive cores, mean ± SD	0.42 ± 0.29
Patients with MRI-visible prostate lesions, *n* (%)	182 (91.5)
Clinical stage, *n* (%)	
T1c	12 (6.0)
T2a	23 (11.6)
T2b	64 (32.2)
T2c	77 (38.6)
≥T3	23 (11.6)

**Table 2:** Gleason grade groups on prostate biopsy and radical prostatectomy.

Gleason grade group at biopsy	Gleason grade group at radical prostatectomy					Total
	3+3 (GR1)	3+4 (GR2)	4+3 (GR3)	8 (GR4)	9-10 (GR5)	
3+3 (GR1)	17	25	8	4	0	54
3+4 (GR2)	2	24	11	6	2	45
4+3 (GR3)	0	5	22	6	4	37
8 (GR4)	0	1	8	14	14	37
9-10 (GR5)	0	0	2	1	23	26
Total	19	55	51	31	43	199

**Table 3:** Multivariable logistic regression models to predict prostate Gleason grade group upgrading.

Predictors	Upgrade		
	OR	95% CI	*p*
Biopsy GR			
GR1	1.000 (reference)		
GR2	0.288	0.122-0.681	0.005
GR3	0.159	0.061-0.414	<0.001
GR4	0.223	0.088-0.564	0.002
GR5	0	0	0.998
PV	0.985	0.970-1.000	0.043
Patient year	1.068	1.013-1.125	0.015
AUC:0.775 (0.712-0.839)			

## Data Availability

The datasets generated and analyzed during the current study are not publicly available due to a series of unfinished studies but are available from the corresponding author on reasonable request.
